# Construction of a prognostic model for HCC based on ferroptosis-related lncRNAs expression and its potential to predict the response and irAEs of immunotherapy

**DOI:** 10.3389/fphar.2023.1090895

**Published:** 2023-03-13

**Authors:** Liangbo Dong, Shengnan Zhou, Xuesong Bai, Xiaodong He

**Affiliations:** ^1^ Department of General Surgery, Peking Union Medical College Hospital (CAMS), Beijing, China; ^2^ Chinese Academy of Medical Sciences and Peking Union Medical College, Dongcheng, China

**Keywords:** ferroptosis, immune checkpoint blockers, immune-related adverse events, lncRNA, heptocellular carcinoma

## Abstract

**Background:** Ferroptosis is an iron-dependent programmed cell death process, and studies have confirmed that it plays an important regulatory role in the occurrence and development of various malignancies including hepatocellular carcinoma (HCC). In addition, the role of abnormally expressed long non-coding RNAs (lncRNAs) in regulating and driving the occurrence and development of HCC has attracted more and more attention. However, there is still a lack of research on the role of ferroptosis-related lncRNAs in the prognosis prediction of HCC patients.

**Method:** In this study, we used the Pearson test method to analyze the association between differentially expressed lncRNAs and ferroptosis-related genes in HCC and normal tissues obtained from The Cancer Genome Atlas (TCGA), and found 68 aberrantly expressed and prognosis-related ferroptosis-related lncRNAs. Based on this, we established an HCC prognostic model composed of 12 ferroptosis-related lncRNAs. In addition, HCC patients were divided into a high-risk group and a low-risk group according to the risk score of this 12 ferroptosis-related lncRNAs prognostic model. Gene enrichment analysis indicated that ferroptosis-related lncRNA-based expression signatures may regulate HCC immune microenvironment signaling pathways through ferroptosis, chemical carcinogenesis-reactive oxygen species, and NK cell-mediated cytotoxicity pathways. In addition, immune cell correlation analysis showed that there were significant differences in immune infiltrating cell subtypes, such as Th cells, macrophages, monocytes, and Treg cells between the two groups. In addition, the expression of multiple immune checkpoint molecules was found to be significantly increased in the high-risk group (eg, PD1, CTLA-4, CD86, etc.).

**Results:** Our research provides a new method for predicting prognosis using a ferroptosis-related lncRNA expression signature prognostic model in hepatocellular carcinoma. And it provides new tools for predicting patient response and adverse effects of immunotherapy.

**Conclusion:** In conclusion, ferroptosis-related lncRNA expression signatures can be used to construct a prognostic prediction model to predict the overall survival of HCC patients, and can be used as an independent influencing factor for prognosis. Further analysis showed that ferroptosis-related lncRNAs may affect the efficacy of immunotherapy in patients with HCC by altering the tumor microenvironment, so this model may serve as a new indicator of the response and irAEs of HCC to immunotherapy.

## 1 Background

Hepatocellular carcinoma (HCC) is the second most common cause of death from human malignancies worldwide and the most common liver malignancy. Llovet et al. (2016) According to research statistics, about 841,000 new cases of hepatocellular carcinoma are diagnosed each year, and about 780,000 patients will die from hepatocellular carcinoma in 2018. Bray et al. (2018) For patients with early-stage hepatocellular carcinoma, local radiofrequency ablation, partial hepatectomy, and liver transplantation are the main treatments, but about 70% of patients will suffer a recurrence within 5 years after surgery. Bray et al. (2018) In recent years, immune checkpoint inhibitors have been proven by many studies to be an effective therapy for the treatment of advanced hepatocellular carcinoma, but their effectiveness still needs to be further improved. Ozer et al. (2021) Although great progress has been made in the early detection and drug treatment of hepatocellular carcinoma, the clinical outcomes of advanced cases are still unsatisfactory. The SEER database shows that the overall 5-year survival rate for hepatocellular carcinoma patients in the United States is 19.6%, while the 5-year survival rate for patients with distant metastases is less than 2.5%. Chidambaranathan-Reghupaty et al. (2021) Due to the high heterogeneity of HCC, there is an urgent need to find new effective molecular markers and improve the prediction accuracy of HCC prognosis to improve the clinical outcomes of HCC and reduce the burden on patients.

Cell death is an essential part of many important physiological and pathological processes in the human body. Vermeulen et al. (2003) Ferroptosis is a relatively new programmed cell death process newly discovered in recent years. It is distinct from other cell death processes such as necrosis, apoptosis, and autophagy. Dixon et al. (2012) Ferroptosis is a form of iron-dependent programmed cell death caused by the accumulation of reactive oxygen species (ROS) generated by lipid peroxidation in cells. Recently, the induction of ferroptosis in tumor cells has become a promising new therapy in the eyes of researchers, especially for malignant tumors that are resistant to conventional radiotherapy and chemotherapy (Liang et al., 2019; Xia et al., 2019; Du et al., 2021; Bekric et al., 2022). With the recent FDA approval of anti-PD-1 or anti-PD-L1 drugs (Keytruda, Tecentriq, nivolumab), immune checkpoint blocker (ICBs) therapy as a new therapy for patients with advanced HCC has gained more and more attention from researchers. Various immune checkpoint inhibitors, alone or in combination with targeted therapy and traditional chemotherapy, are also increasingly used to treat patients with advanced hepatocellular carcinoma (Pinter et al., 2021a; Pinter et al., 2021b; Wu et al., 2022). However, only part of these patients can benefit from immunotherapy, possibly due to the complexity and heterogeneity of the tumor itself, as well as many unknown factors in the tumor microenvironment (TME) (El Dika et al., 2019; Lee et al., 2020; Zhu et al., 2022). The complex tumor microenvironment may reduce the efficacy of immunotherapy, and the underlying mechanism may be related to various stromal cells and various types of immunosuppressive factors contained in the microenvironment. Prieto et al. (2015) Therefore, it is crucial to further explore novel molecular mechanisms in HCC and develop a new indicator to evaluate the response of HCC patients to immunotherapy, thereby optimizing the treatment strategy. A recent study found that CD8^+^ T-cells induced by immunotherapy could enhance ferroptosis by altering the microenvironment and releasing cytokines, thereby reducing the expression level of SLC7A11 in tumor cells to suppress the tumor Wang et al. (2019). This suggests a relationship between the ferroptosis process in tumor cells and immune system activation. Another study has shown that tumor cells with ferroptosis may act as donor cells to produce biologically active immunomodulatory arachidonic acid metabolites to affect anti-tumor immunity Friedmann Angeli et al. (2019). Therefore, it is necessary to study tumor immunotherapy from the perspective of the ferroptosis mechanism. A large number of experimental studies have also shown that ferroptosis-related genes play a crucial role in the occurrence and development of hepatocellular carcinoma (Sun et al., 2016; Liao et al., 2021; Wang et al., 2021; Yao et al., 2021).

Long non-coding RNAs (lncRNAs) are self-transcribed non-coding RNAs with a minimum fragment length of about 200 nucleotides, which can participate in various complex biological processes (Cech and Steitz, 2014; Quinn and Chang, 2016). Previous studies have shown that lncRNAs are abnormally expressed in a variety of malignant tumors, and other studies have shown that abnormally expressed lncRNAs can be used as prognostic indicators for various malignancies including hepatocellular carcinoma (Xu et al., 2022a; Xu et al., 2022b; Cui et al., 2022; Zhou et al., 2022; Zhu et al., 2022). By interacting with proteins or DNAs, lncRNAs play important roles in the occurrence and progression of different types of tumors, including HCC Huang et al. (2020). However, studies on ferroptosis-related lncRNAs related to the prognosis of HCC patients are still insufficient. Therefore, this study aimed to establish a novel prognostic model of ferroptosis-related lncRNAs expression signature to predict the prognosis of HCC patients and to improve the current diagnosis, treatment, follow-up, and prevention of HCC.

In the present study, we identified the expression signatures of lncRNAs associated with ferroptosis in hepatocellular carcinoma by correlation analysis and constructed a new prognostic model based on 12 ferroptosis-associated lncRNAs using multivariate Cox regression analysis. Then we assessed the ability of this model to independently predict the prognosis of HCC patients and explored the role of ferroptosis-related lncRNAs in tumor immunity. In conclusion, this study found that ferroptosis-related lncRNA can affect the efficacy of immunotherapy by affecting immune cell infiltration in the tumor microenvironment, so it has the potential to serve as an ideal biomarker for evaluating the therapeutic effect and adverse effects of immunotherapy.

## 2 Methods

### 2.1 Data and information collection

In this study, the transcriptome RNA sequencing (RNA-seq) data of 371 hepatocellular carcinoma patients with complete clinical data were downloaded from the TCGA official website (http://portal.gdc.cancer.gov/). This study normalized the mRNA expression data for each patient using an algorithm provided by the R package (Limma). The corresponding clinical and pathological characteristics of the enrolled patients, including age, gender, tumor differentiation, TNM stage, survival time, and survival status, were also downloaded from the TCGA database. The data involved in the TCGA database are publicly available, therefore, this study does not require ethics committee approval.

### 2.2 Identification of lncRNAs associated with the ferroptosis

The FerrDb database is the first manually organized ferroptosis database established by Chinese researchers. The database includes ferroptosis-related driver and suppressor genes, markers, various regulatory factors, and ferroptosis-related disease data. In this study, ferroptosis-related genes were retrieved from the FerrDb (http://www.zhounan.org/ferrdb/) database. A total of 382 ferroptosis-related genes were finally included. Relationships between lncRNAs and ferroptosis-related genes were calculated based on RNA expression levels. Co-expression analysis was performed using Spearman’s correlation coefficient to identify lncRNAs related to ferroptosis. The absolute value > 0.4, and the *p*-value < 0.001 were defined as ferroptosis-related lncRNAs.

### 2.3 Construction and validation of ferroptosis-related lncRNA prognosis prediction model

Firstly, lncRNA expression and clinical data were analyzed. Ferroptosis-related lncRNAs associated with prognosis were identified using univariate Cox regression. Then, ferroptosis-related lncRNAs with *p* values ≤0.05 were included in multivariate Cox regression to construct a prognostic prediction model based on the expression of ferroptosis-related lncRNAs. The risk score formula used in the prognostic model is as follows: risk score = e^sum (lncRNA expression ×corresponding coefficient)^. Patients were separated into low-risk or high-risk groups based on the median value. Differences in survival status between the two risk groups were assessed by Kaplan-Meier (KM) and tested with the log-rank test method. The ROC curve and calibration curve were used to determine the accuracy of the prognostic prediction model. Then, combined with other clinical characteristics of the enrolled patients, it was determined whether the prognostic prediction score could be used as an independent influencing factor of prognosis, and a nomogram was drawn below.

### 2.4 Functional enrichment analysis of related lncRNA genes

In this study, the R package (ClusterProfiler) was used to perform GO enrichment analysis (Gene Ontology, GO) and KEGG enrichment analysis (Kyoto Encyclopedia of Genes and Genomes, KEGG). *p* values were still adjusted by the BH method. Gene set enrichment analysis (GSEA) in the R package (gsva) was used to investigate functional phenotypic differences between two risk groups (high-risk group and low-risk group). In this study, we functionally enriched ferroptosis-related lncRNAs and visualized the pathways that are closely related to immunity and tumorigenesis and development. The gene sets used were downloaded from the Molecular Signatures database and analyses were run in GSEA software (version 4.2.3). *p* values <0.05 and FDR <0.05 were considered statistically significant.

In the statistical analysis of this study, all *p* values were two-tailed and *p* < 0.05 was considered statistically significant. Kaplan-Meier survival curves were used to compare survival differences between different risk groups (low-risk and high-risk groups). Univariate and multivariate Cox regression analyses were used to identify independent clinical prognostic factors. In the GSEA analysis comparing immune cells and immune-related functions between the two groups, the differences in scores were tested by the Mann-Whitney test. All statistical analyses were performed in R software (version 4.1.3). Relevant R packages used in the study include ggplot2, stats, Rtsne, timeROC, glmnet, gsva, survival, and survminer, etc.

## 3 Results

### 3.1 lncRNAs associated with ferroptosis in hepatocellular carcinoma

A list of 382 ferroptosis-related genes was first extracted and downloaded from the FerrDb database. And lncRNAs with significant correlation with ferroptosis genes were found through co-expression analysis. The filter condition was set to the correlation coefficient Cor>0.4, and the *p*-value < 0.001. After co-expression analysis, 1,278 lncRNAs related to ferroptosis were obtained. The obtained ferroptosis-related lncRNAs were further used in a univariate Cox proportional hazards regression model to find out which lncRNAs were associated with prognosis.

### 3.2 Construction and validation of ferroptosis-related lncRNA prognosis prediction model

**FIGURE 1:**
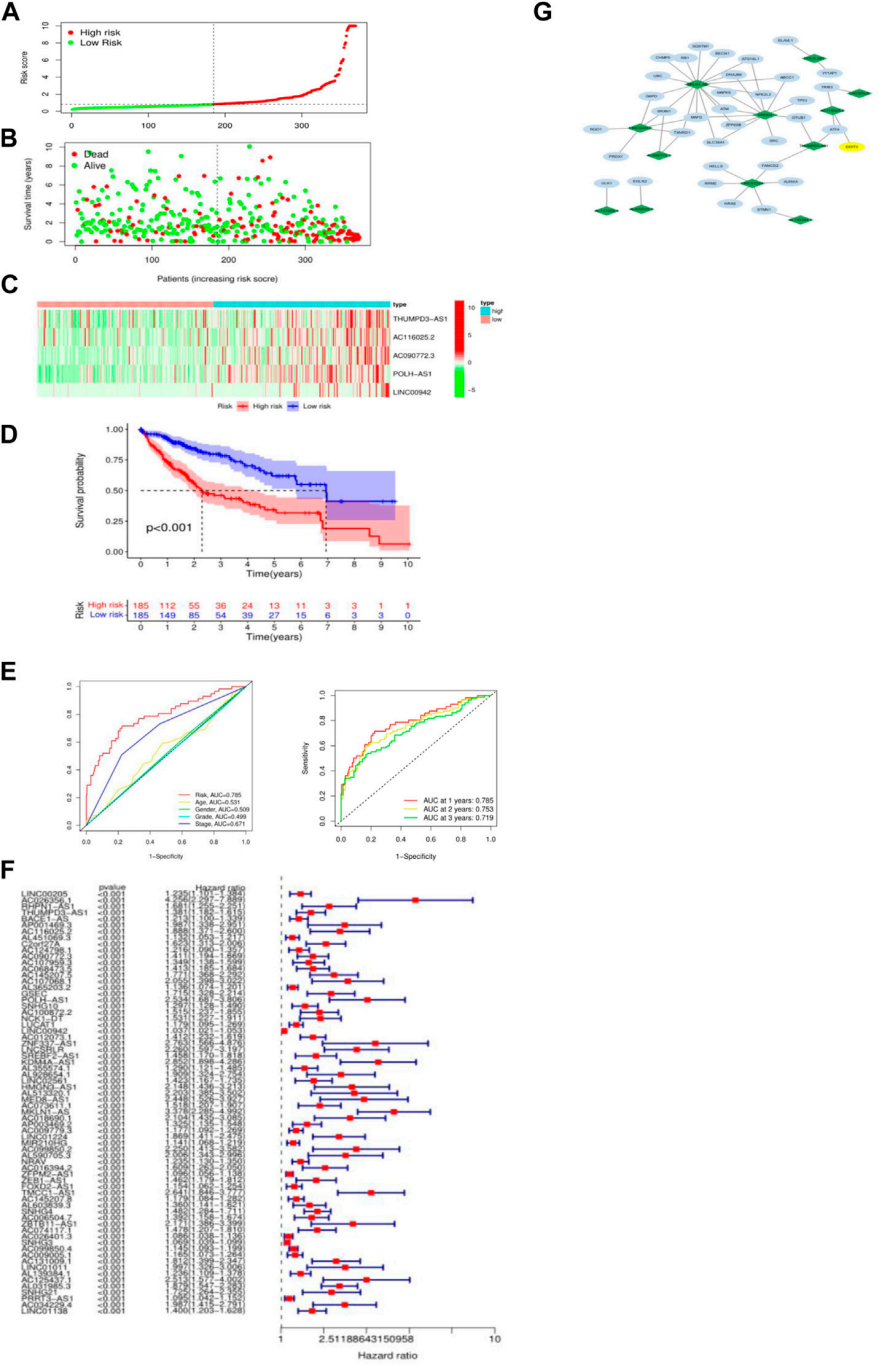
**(A)** Risk score distribution and median value in TCGA HCC cohort; **(B)** Survival status, overall survival time (OS), and risk score distribution of HCC patients in TCGA cohort; **(C)** Heatmap of expressions of 12 selected ferroptosis-related lncRNAs in high-risk and low-risk groups; **(D)** Kaplan-Meier survival curves of two groups of patients (high-risk group and low-risk group); **(E)** The AUC for risk score and clinical features according to the ROC curves, and ROC curve analysis within 1, 2, and 3 years. **(F)** Ferroptosis-related lncRNA expression and overall survival Forest plot of univariate Cox regression analysis of period (OS); **(G)** Schematic diagram of ferroptosis-related lncRNA and mRNA correlation network (diamond is lncRNA, oval is ferroptosis-related gene mRNA).

Combined analysis of ferroptosis-related lncRNAs and survival data, using univariate Cox regression, the analysis showed that 68 ferroptosis-related lncRNAs were closely related to overall survival (OS) in patients with hepatocellular carcinoma, and the high expressions of these lncRNAs were associated with poor prognosis. (Figure 1F). With LASSO Cox regression analysis, finally, 12 lncRNAs related to ferroptosis were screened, and based on their expression data, a prognostic prediction model for hepatocellular carcinoma patients was constructed. The calculation formula in the model is: Risk Score = 0.288 × THUMPD3-AS1 expression +0.538 × AC116025.2 expression +00.201 × AC090772.3 expression +0.797 × POLH-AS1 expression +0.031 × LINC00942 expression +0.695 × LNCSRLR expression +0.785×MKLN1-AS expression +0.302×LINC01224 expression +0.277 × AL603839.3 expression +0.332×SNHG4 expression +0.411 × AC131009.1 expression +0.214 × AL139384.1 expression. The hazard ratio of each lncRNA to survival time (OS) in this model was greater than 1, and the expression in HCC tissue was significantly higher than that in normal liver tissue. Patients were classified into a high-risk group (n = 185) and a low-risk group (n = 185) according to the median TCGA group risk score (0.828). The distribution of risk scores of the two groups is shown in the figure (Figure 1A), and the distribution of survival status of patients also shows that the overall survival of patients in the high-risk group is significantly shortened in the lower-risk group (Figure 1B). The heatmap of lncRNA expression involved in the construction of the prognostic prediction model showed that all 12 ferroptosis-related lncRNAs were highly expressed in the high-risk group (Figure 1C). The Kaplan-Meier survival curves of the two groups of patients showed that the overall survival (OS) of patients in the high-risk group was significantly lower than that of the patients in the low-risk group (*p* < 0.0001) (Figure 1D).

The predictive performance of the model in predicting the overall survival (OS) risk score was then evaluated by time-dependent ROC curves, with AUC reaching 0.785 at 1 year, 0.753 at 2 years, and 0.719 at 3 years (Figure 1E). To further analyze the interaction between 12 ferroptosis-related lncRNAs and ferroptosis-related gene expression, Cytoscape software was used to visualize the co-expression network of lncRNAs and mRNAs. Death-related genes are at the center of the correlation network. (Figure 1G).

### 3.3 The independent prognostic value of this prediction model based on 12 ferroptosis-related lncRNA expression signatures

**FIGURE 2:**
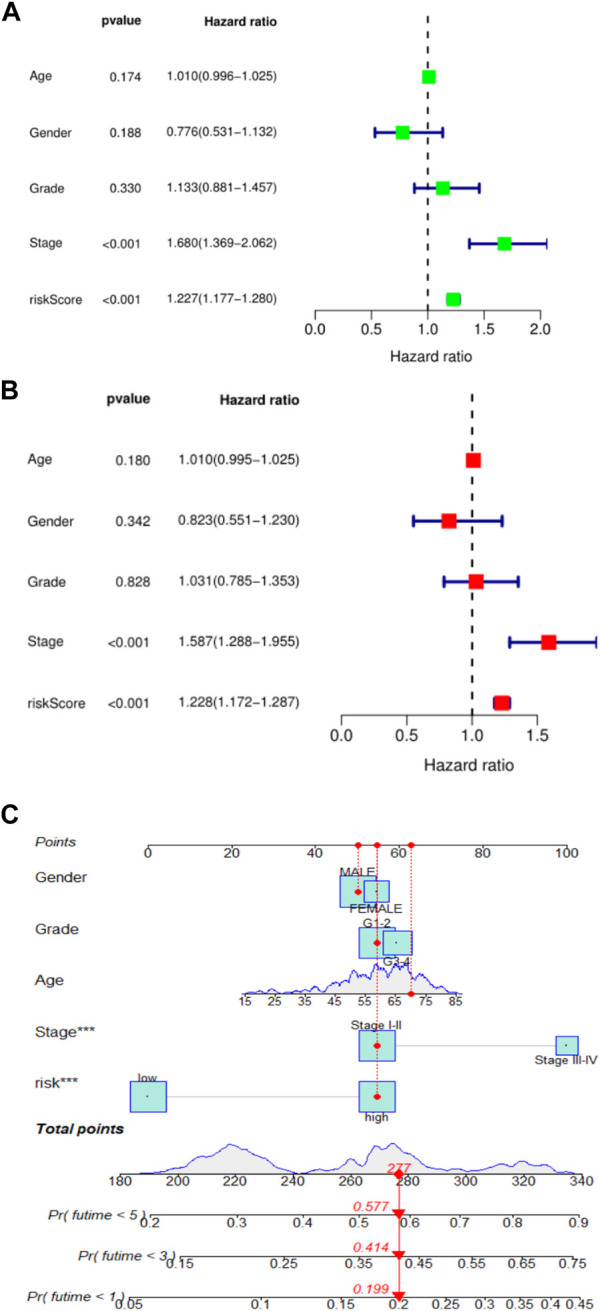
**(A)**. Univariate Cox regression analysis of risk score and survival in TCGA cohort; **(B)**. Univariate Cox regression analysis of risk score and survival in TCGA cohort; **(C)**. nomogram of 1, 3, and 5-year survival rates of liver cancer patients.

To further validate the prognostic value of this risk score model, we performed univariate and multivariate Cox regression analyses using patient age, sex, tumor grade, TNM stage, and risk score as variables. Results could determine whether risk score can be used as an independent prognostic predictor of overall survival (OS). In univariate Cox regression analysis, the risk score of the TCGA cohort was significantly associated with overall survival (OS) (HR = 1.227, 95% CI = 1.177–1.280, *p* < 0.001) (Figure 2A). After adjusting for other confounding factors, risk score remained an independent predictor of overall survival (OS) in multivariate Cox regression analysis (HR = 1.228, 95% CI = 1.172–1.287, *p* < 0.001) (Figure 2B). These results confirmed that this new HCC patient prognosis prediction model based on ferroptosis-related lncRNA expression signature can be reliably used as a novel tool for HCC patient prognosis prediction. To make the prognostic prediction model based on ferroptosis-related lncRNA more applicable to the clinic, this study also established a nomogram to better help clinicians to predict the 1-, 3-, and 5-year survival rates of patients. The predictors in the nomogram included the risk score and other clinicopathological characteristics (age, gender, tumor grade, tumor stage) of the predictive model (Figure 2C). In the plotted nomogram, the risk score model exerted excellent weights in all of these clinically relevant variables, which is also consistent with the results of the multivariate Cox regression analysis. These results collectively confirm that this novel lncRNA prediction model associated with ferroptosis can reliably serve as an independent prognostic factor in HCC patients.

### 3.4 Functional enrichment analysis of ferroptosis-related genes

**FIGURE 3:**
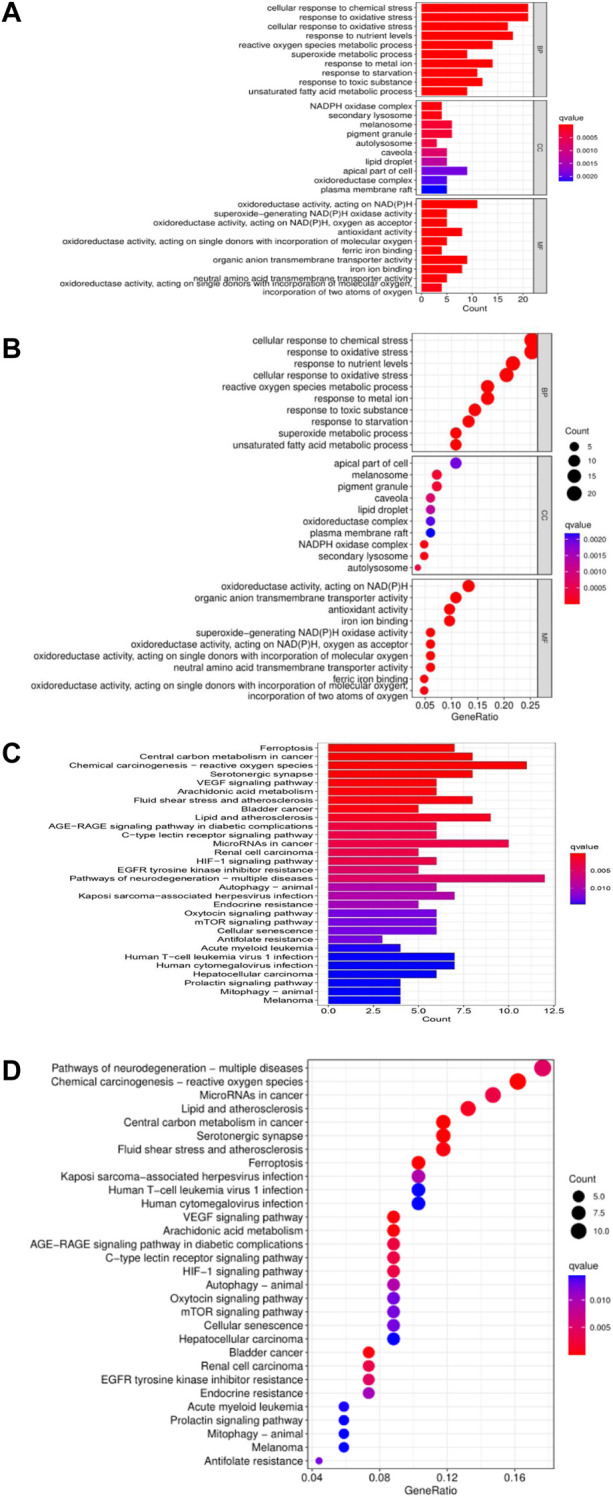
GO enrichment analysis of ferroptosis-related lncRNAs in the TCGA liver cancer cohort **(A, B)** and KEGG enrichment analysis **(C, D)**.

To further understand the molecular mechanism of ferroptosis-related differentially expressed genes and how it affects the occurrence and development of hepatocellular carcinoma, GO enrichment and KEGG enrichment analysis were also performed in this study. GO enrichment analysis showed that: in the RNA-seq expression data of TCGA HCC patients, in terms of biological processes, it can be observed that related genes are enriched in various cellular stress response processes, such as cellular oxidative stress, cellular chemical stress, etc. Consistent with the expected results, there were significant enrichment phenomena in the intracellular redox reaction chain and iron metabolism, including a variety of enzymes involved in NADPH oxidation, antioxidant reaction processes, iron ion binding, transmembrane transporters, the redox reaction of molecular oxygen, etc. KEGG enrichment result was also as predicted before, these genes were enriched in ferroptosis, chemical carcinogenesis process - reactive oxygen species (ROS), superoxidation process, mTOR signaling pathway, and autophagy-related signaling pathway. And it is related to a variety of malignant tumor-related pathways, including acute myeloid leukemia-related pathways, renal cell carcinoma, and bladder cancer-related pathways. In addition, it can be observed that these genes are enriched in the EGFR tyrosine receptor signaling pathway, the VEGF receptor pathway, etc., which also implies that ferroptosis may play a certain role in the targeted therapy of hepatocellular carcinoma. (Figure 3).

**FIGURE 4:**
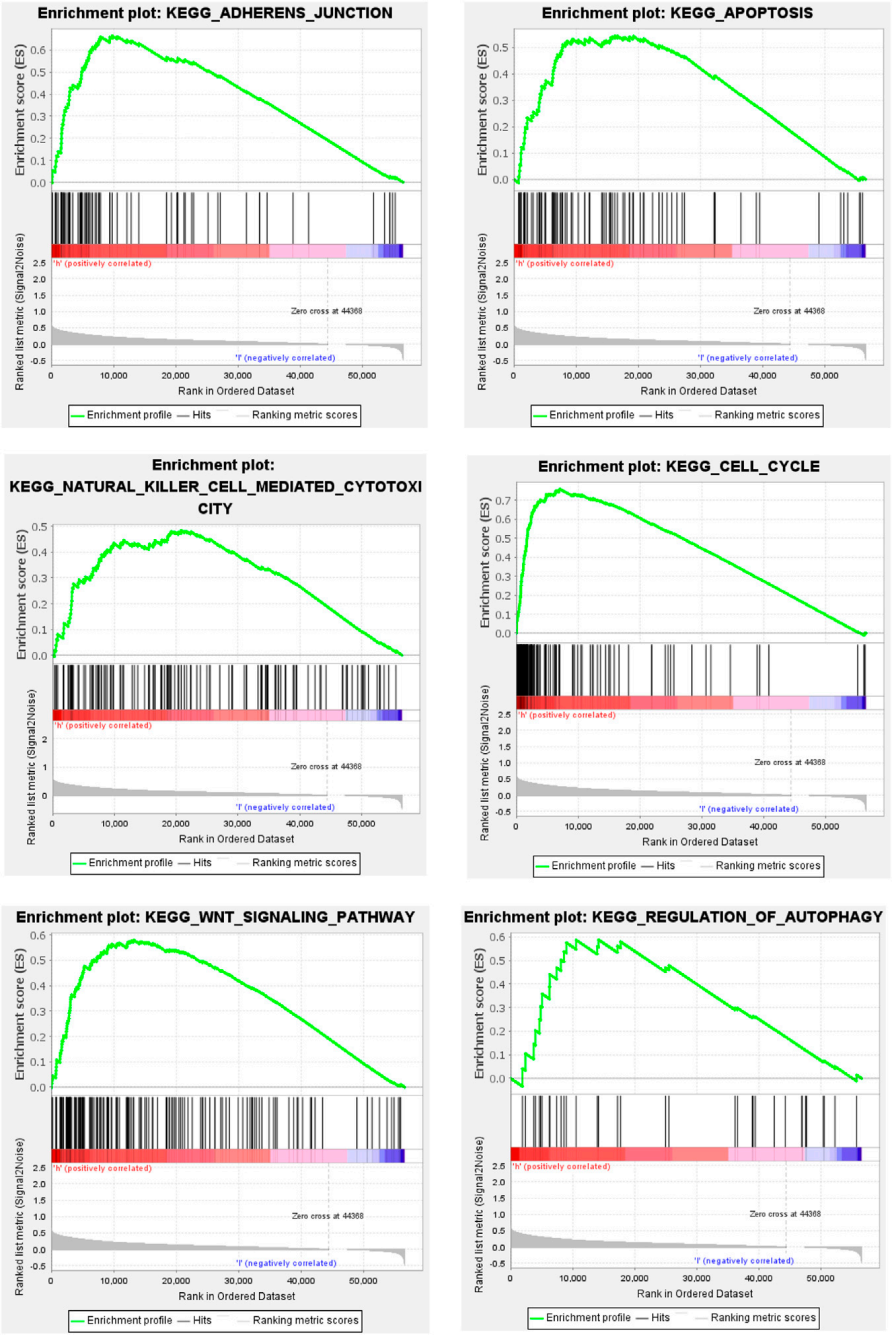
Partial GSEA enrichment analysis results.

To further explore the mechanism of ferroptosis-related lncRNA in the occurrence and development of hepatocellular carcinoma. We performed GSEA analysis, and the results showed that the enrichment of gene sets in high-risk group patients included cell adhesion pathway, apoptosis pathway, cell cycle pathway, DNA replication, endocytosis, fatty acid metabolism, insulin receptor pathway, and mTOR-like receptors. In addition, some immune-related pathways were also significantly enriched in the high-risk group, including B-cell receptor (BCR), T-cell receptor (TCR), NK cell-mediated cytotoxic effector pathways, etc. These results suggest that patients with high-risk scores in this predictive model may be associated with enhanced DNA replication, abnormal metabolic pathways, activation of some classical tumor signaling pathways, and tumor immune escape (Figure 4).

### 3.5 Relationship between ferroptosis-related lncRNAs and tumor-infiltrating cells in hepatocellular carcinoma

**FIGURE 5:**
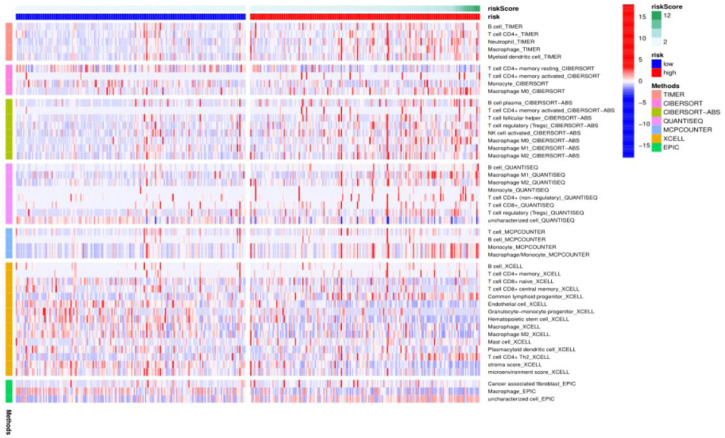
Heat map of immune cell correlation analysis in TCGA HCC cohort ferroptosis-related lncRNA prognostic model.

To further explore the mechanism of ferroptosis-related lncRNAs involved in the occurrence and development of hepatocellular carcinoma, we used the algorithms of CIBERSORT, CIBERSORT-ABS, XCELL, EPIC, MCPCOUNTER, QUANTISEQ and TIMER to draw a heat map of immune cell correlations as shown below. It was found that some immune-infiltrating cells, TICs, including dendritic cells, neutrophils, macrophages, mast cells, monocytes, and regulatory T (Treg) cells were enriched in the high-risk group significantly higher than in the low-risk group. These findings strongly suggest that our selected ferroptosis-related lncRNA expression signature is significantly associated with immune cell infiltration in HCC (Figure 5).

### 3.6 Correlation between ferroptosis-related lncRNA signatures and ICB treatment outcomes and irAEs

**FIGURE 6:**
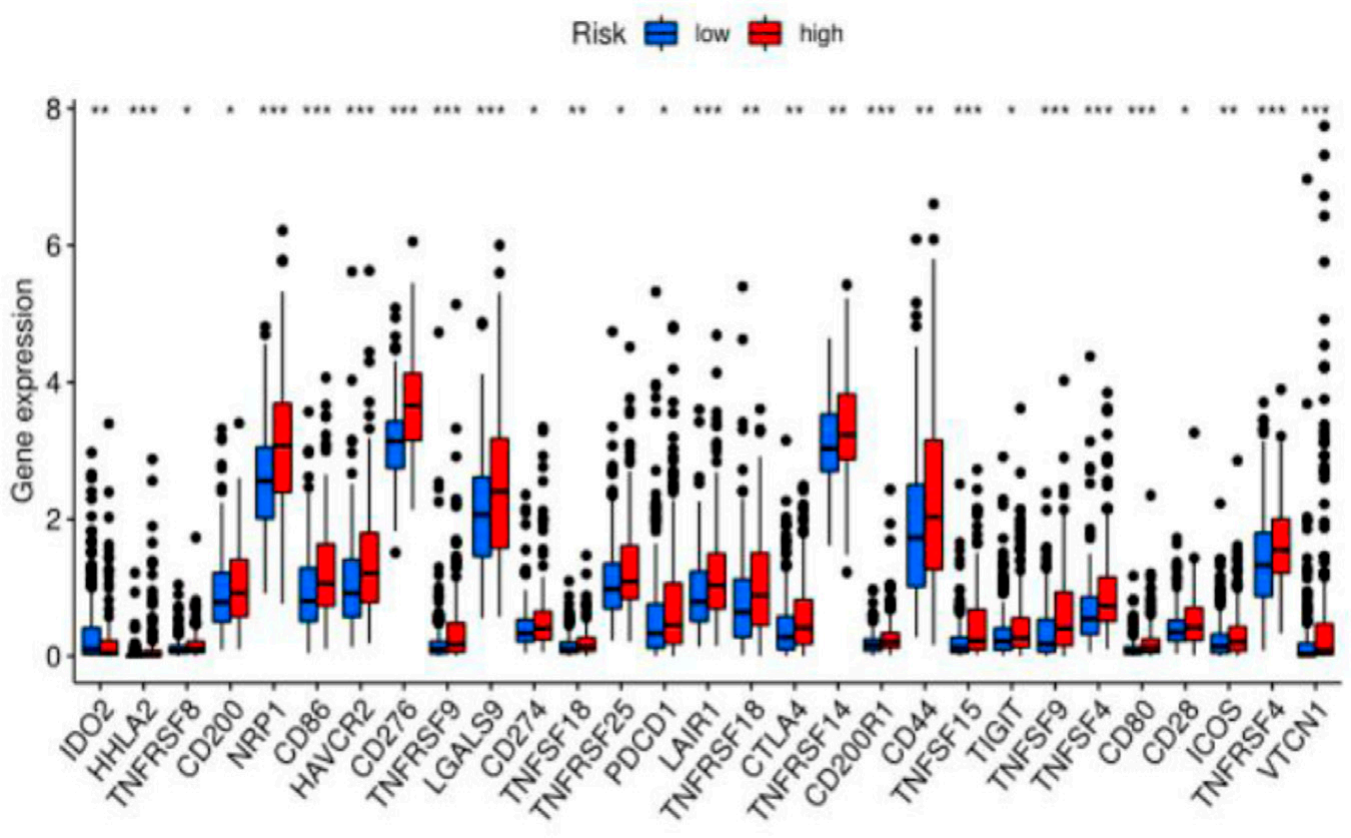
Difference analysis of immune checkpoints between two groups (high-risk group and low-risk group) in the TCGA cohort.

Previous sections suggested a correlation between ferroptosis-related lncRNAs, tumor-infiltrating cells, and immunological signatures. Based on these findings, we further investigated the role of these lncRNAs in immunotherapy treatment and adverse effects. We found that the expression levels of the above ferroptosis-related lncRNAs were significantly correlated with immune checkpoint gene expression (PD-1 (*p*-value <0.05), CTLA-4 (*p*-value <0.05), IDO2 (*p*-value <0.05), CD44 (*p*-value <0.05), LAG3 (*p*-value <0.05)). This suggests that abnormally high expression of immune checkpoint proteins can be observed in patients in the high-risk group. Some of these proteins were also identified as an independent predictor of irAEs (immune-related adverse events) development. This suggests that patients in the high-risk group may benefit from immunotherapy and have a greater chance of developing irAEs (Figure 6).

## 4 Discussion

Hepatocellular carcinoma is one of the most common malignant tumors in the world with a high mortality rate. Due to the heterogeneity of the tumor itself, it is extremely difficult for clinicians to predict the prognosis of patients. Therefore, it is very important to develop a reliable and effective prognostic biomarker for HCC. In this study, we developed a novel prognostic model based on 12 ferroptosis-related lncRNA expression signatures in the TCGA HCC cohort, and it shows good prediction performance.

Studies have shown that lncRNAs play a key role in the chromatin structure, cell growth, gene expression, differentiation, and development of human cells, and their abnormal expression or mutation is closely related to a variety of diseases, especially malignant tumors (Peng et al., 2017; Sato et al., 2021; Wu et al., 2021). It is believed that lncRNAs are associated with multiple malignant tumor-related processes, such as proliferation, invasion, migration, and angiogenesis (Schmitt and Chang, 2016). For the treatment of hepatocellular carcinoma, lncRNAs can be used as biomarkers to predict the efficacy of patients receiving surgery, radiotherapy, chemotherapy, and immunotherapy, and it is expected to become an important tool for individualized diagnosis and treatment of hepatocellular carcinoma. Yuan et al. (2021) In existing studies, many scholars have used a variety of lncRNA expression features to predict the prognosis of various malignant tumors and constructed different prognostic models, including breast cancer, colorectal cancer, lung cancer, gastric cancer, bladder cancer, etc. (Liu et al., 2020; Ma et al., 2020; Shen et al., 2020; Song et al., 2021; Xu et al., 2021). In HCC, other researchers have developed a variety of lncRNA expression signature-based prognostic prediction models based on differentially expressed lncRNAs and certain tumor pathogenesis. For example, the 11 lncRNAs (AC010547.1, AC010280.2, AC015712.7, GACAT3, AC079466.1, AC089983.1, AC051618.1, AL121721.1, LINC01747, LINC01517, and AC008750.3) expression signatures can be used to effectively predict the risk of death from hepatocellular carcinoma (Li et al., 2020). Another study expression signatures also constructed a liver cancer prognosis model using seven autophagy-related lncRNAs (PRRT3-AS1, RP11-479G22.8, RP11-73M18.8, LINC01138, CTD-2510F5.4, CTC-297N7.9, RP11-324I22.4) and demonstrated good predictive performance (Yang et al., 2021). In addition, the biological functions of selected lncRNAs in hepatocellular carcinoma have been confirmed in multiple independent studies, for example, MKLN1-AS can affect HCC epithelial-mesenchymal transition (EMT) through the SOX9-MKLN1-AS axis, which promoted the proliferation and migration of hepatocellular carcinoma cells (Guo et al., 2022). LINC01224 could downregulate the expression of CHEK1 through competitive binding with miR-330-5p, thereby inhibiting the progression of hepatocellular carcinoma (Gong et al., 2020).

With the development of immune checkpoint inhibitors, immunotherapy as an emerging therapy has shown a considerable therapeutic effect on hepatocellular carcinoma (Foerster et al., 2022; Llovet et al., 2022). Currently, immunotherapy combined with anti-angiogenic targeted therapy provides a new promising treatment strategy for advanced liver cancer. However, more than two-thirds of patients still show an unsatisfied response to immunotherapy (Mushtaq et al., 2018). A recent study showed that ferroptosis combined with immune checkpoint inhibitors can synergistically enhance antitumor activity, a phenomenon seen even in immunotherapy-resistant tumors (Tang et al., 2020). Therefore, a new predictive model based on the expression characteristics of ferroptosis-related lncRNAs can be considered to study the relationship between immunotherapy and ferroptosis and predict the efficacy of immunotherapy. In our study, we found that the expression signature of the lncRNAs we selected was related to the expression of immune checkpoint proteins (i.e., PD-1, CTLA-4 and CD28, etc.). This suggests that the model could potentially be used to predict patients’ responses to immunotherapy. Meanwhile, the expression levels of these immune checkpoint proteins were higher in the high-risk group than in the low-risk group. This indicates that the expression signature of ferroptosis-related lncRNAs can be used to predict the expression level of immune checkpoint proteins in tumor tissues, and has the potential to be seen as a new indicator to guide immunotherapy decisions. With the wide application of ICBs in the treatment of HCC, the toxic and side effects caused by the activation of the immune system by ICI, which is, immune-related adverse events, have become a major challenge in clinical practice (Postow et al., 2018). There are no validated biomarkers to predict the irAEs before ICBs treatment until now. Some genes are associated with irAEs, and the expression levels of these genes were higher in the high-risk group in our study. It suggests that our model has the potential to predict the occurrence of irAEs. But these findings still need to be proved in larger studies, and multi-omics prediction could have better performance (Jing et al., 2020; Wölffer et al., 2022). In addition, this study also showed that the risk score of ferroptosis-related lncRNAs expression signature was associated with immune infiltrating cells (B-cells, macrophages, myeloid dendritic cells, neutrophils, and CD4^+^ T-cells) in HCC tissues, which means that this prognostic model may play an important role in immune infiltration.

However, our study still has some limitations. This study is primarily a retrospective study based on comprehensive bioinformatics analysis and public database data, and these findings lack solid clinical validation. In addition, the accuracy of the ferroptosis-related lncRNA expression signature prognostic model for the immune regulation of HCC patients will remain an important clinical issue, which needs to be verified by prospective experiments.

## Data Availability

The original contributions presented in the study are included in the article/supplementary material, further inquiries can be directed to the corresponding author.
